# Fever in Ireland

**Published:** 1847-04

**Authors:** 


*Fever in Ireland.*—Fever is rapidly extending its ravages even in Dublin. The Cork street hospital, one of the largest establishments of its kind in Ireland, is literally crammed with patients, to such a degree of inconvenience, indeed, that the governors have given directions to have temporary buildings—if sheds or tents can be so called—prepared for the reception of the numerous patients for whom there is no accommodation within doors. The state of the Meath and Richmond hospitals is equally deplorable, and the accounts from all parts of the country represent disease and destitution proceeding at an equal pace.—*Medical Times.*

